# Construction of an immunotoxin via site-specific conjugation of anti-Her2 IgG and engineered *Pseudomonas* exotoxin A

**DOI:** 10.1186/s13036-019-0188-x

**Published:** 2019-06-21

**Authors:** Byeong Sung Lee, Yumi Lee, Jisoo Park, Bo Seok Jeong, Migyeong Jo, Sang Taek Jung, Tae Hyeon Yoo

**Affiliations:** 1 Department of Molecular Science and Technology, Ajou University, 206 World cup-ro, Yeongtong-gu, Suwon, 16499 South Korea; 2 Department of Applied Chemistry and Biological Engineering, Ajou University, 206 World cup-ro, Yeongtong-gu, Suwon, 16499 South Korea; 3 Department of Applied Chemistry, Kookmin University, 77 Jeongneung-ro, Seongbuk-gu, Seoul, 02707 South Korea; 4 Department of Biomedical Sciences, Graduate School of Medicine, Korea University, Seongbuk-gu, Seoul, 02841 South Korea

**Keywords:** Immunotoxin, Site-specific conjugation, Immunoglobulin G, Unnatural amino acid, *Pseudomonas* Exotoxin A

## Abstract

**Background:**

Immunotoxins consisting of a toxin from bacteria or plants and a targeting module have been developed as potent anti-cancer therapeutics. The majority of them, especially those in preclinical or clinical testing stages, are fusion proteins of a toxin and antibody fragment. Immunotoxins based on full-length antibodies are less studied, even though the fragment crystallizable (Fc) domain plays an important role in regulating the concentration of immunoglobulin G (IgG) in the serum and in antibody-mediated immune responses against pathogens.

**Results:**

We devised a method to site-specifically conjugate IgG and another protein using a cysteine residue introduced into the IgG and a bio-orthogonally reactive unnatural amino acid incorporated into the other protein. The human epidermal growth factor receptor 2 (Her2)-targeting IgG, trastuzumab, was engineered to have an unpaired cysteine in the heavy chain, and an unnatural amino acid with the azido group was incorporated into an engineered *Pseudomonas* exotoxin A (PE24). The two protein molecules were conjugated site-specifically using a bifunctional linker having dibenzocyclooctyne and maleimide groups. Binding to Her2 and interaction with various Fc receptors of trastuzumab were not affected by the conjugation with PE24. The trastuzumab-PE24 conjugate was cytotoxic to Her2-overexpressing cell lines, which involved the inhibition of cellular protein synthesis due to the modification of elongation factor-2.

**Conclusions:**

We constructed the site-specifically conjugated immunotoxin based on IgG and PE24, which induced target-specific cytotoxicity. To evaluate the molecule as a cancer therapeutic, animal studies are planned to assess tumor regression, half-life in blood, and in vivo immunogenicity. In addition, we expect that the site-specific conjugation method can be used to develop other antibody-protein conjugates for applications in therapeutics and diagnostics.

**Electronic supplementary material:**

The online version of this article (10.1186/s13036-019-0188-x) contains supplementary material, which is available to authorized users.

## Background

Strategies to chemically or physically link a cytotoxic moiety to a targeting molecule have enabled the development of efficacious therapeutics with reduced side effects. These include immunotoxins [1–3] composed of bacteria- or plant-derived toxins, and antibodies or ligands targeting receptors on cell surfaces. The cytotoxicity of some immunotoxins is induced by the enzymatic reactions of toxins [4, 5], which could result in greater efficacy than that of other antibody-based cytotoxic molecules. In addition, the unique cell-killing mechanism of immunotoxins could enable the development of therapies in combination with other therapeutic molecules [6–8].

One of the intensively studied immunotoxins is a fusion protein composed of an antibody fragment, such as variable fragment (Fv), single-chain Fv (scFv), or antigen binding fragment (Fab), and an engineered *Pseudomonas* exotoxin A (PE) [3, 9]. After endocytosis via interaction with a ubiquitous cell-surface receptor (CD91), the toxin translocates into the cytoplasm and then ADP ribosylates eukaryotic elongation factor-2 (eEF-2) [10, 11]. The modified eEF-2 displays defects in GDP-GTP exchange, GFP hydrolysis, and binding to pre-translocation ribosome, which results in inhibition of the protein synthesis and finally cellular death [12]. Immunotoxins have been developed by replacing the domain of PE interacting with CD91 with antibody fragments. For example, an anti-CD22 immunotoxin recombinant designated moxetumomab pasudotox has recently been approved for the treatment of hairy cell leukemia [13]. To improve the efficacy and the toxicity of PE, portions of the toxin that were not necessary for the translocation and the modification of eEF-2 were deleted, resulting in PE24, which showed much lower off-target toxicity than the original [14], and its immunogenicity was reduced by removing T- and B-cell epitopes [15, 16].

Antibody fragments can offer several advantages over full-length antibodies. These include lower production costs because of the use of microbial expression systems, higher tumor penetration efficiency due to their smaller sizes, and potentially reduced immunogenicity [17]. On the other hand, the absence of the fragment crystallizable (Fc) domain and/or their small size results in a much shorter half-life than full-length antibodies as well as the lack of interactions with the immune system [17]. For example, Benhar and coworkers reported a method to prepare a complex of an anti-Her2 IgG and a fusion protein of an Fc-binding protein, which they termed the Z domain, and an engineered PE [18]. The immunotoxin displayed higher efficacy in a mouse xenograft model than the recombinant form of an anti-Her2 Fv and the PE protein. In particular, the in vivo half-life of the complex form was 240 min in the mouse model, which was approximately 13-fold longer than the recombinant form. However, the interaction between IgG and PE is reversible because it depends on the noncovalent bond between Fc and the Z domain, and PE dissociated from the immunotoxin loses its ability to target disease-causing cells, which might be associated with side effects. Recently, the advantages of extending the half-life of an immunotoxin were demonstrated by constructing an engineered immunotoxin having an albumin-binding domain [19]; albumin and agents bound to albumin have a long half-life in circulation [20, 21].

Recombinant fusion of immunoglobulin G (IgG) and PE has not been successful so far, possibly because of the toxicity of PE expressed in eukaryotic cells. Non-specific chemical crosslinking between PE and IgG was tried to prepare a PE-based immunotoxin [22]. However, the products were heterogeneous, which could be a critical hurdle to develop them as therapeutics. Two methods have been developed to specifically conjugate IgG and PE: intein-mediated ligation [23] and peptide-directed photo-crosslinking [24]. However, despite the successful production of conjugates, these methods have limitations in choosing positions in IgG or toxin for conjugation. In addition, relatively large fusions to IgG and PE are needed for conjugation in the intein-mediated ligation, which might not be applicable to other pairs. The peptide-directed photo-crosslinking method results in blocking of at least one of the two FcRn-binding sites of IgG. This is undesirable because the interaction between Fc and FcRn plays an important role in the long half-life of IgG.

As an alternative, we turned to a conjugation method based on an engineered cysteine (Cys) residue in IgG and a bio-orthogonally reactive unnatural amino acid introduced into PE. The conjugation method based on Cys has been used to generate antibody-drug conjugates [25–29] and several molecules developed using this technology are presently in clinical trials [30, 31]. Using this strategy allows us to leverage an established technology to manufacture new therapeutics. It also allows us to systematically investigate conjugation at different sites in the IgG [32]. Methods have been developed to incorporate unnatural amino acids at intended positions of protein [33–35], and these abiological groups have allowed site-specific conjugation of proteins with various molecules [36–40].

**Scheme 1:**
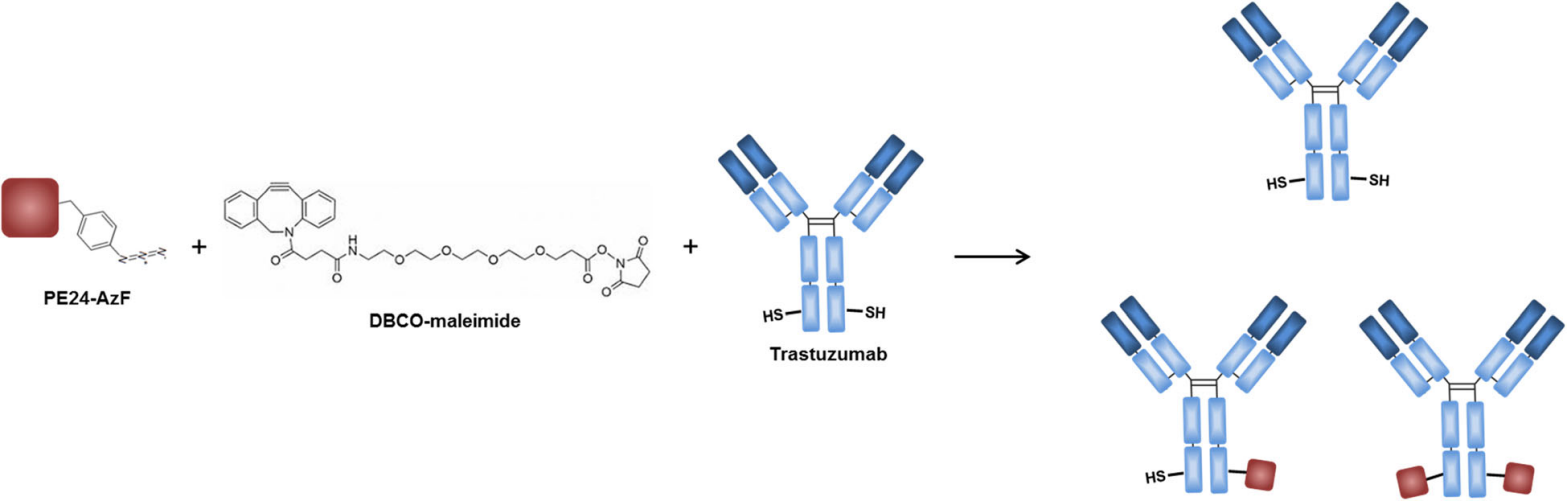
Conjugation of trastuzumab and PE24-AzF. Trastuzumab, Her2-targeting IgG, was engineered with an unpaired Cys, and an unnatural amino acid having the azido group was incorporated into PE24 to install the bioorthogonal reactive moiety at the toxin protein. The two proteins were conjugated via a linker having bifunctional groups of maleimide and dibenzocyclooctyne (DBCO), which are reactive with the thiol and azido groups, respectively. The introduced Cys residues are shown in the heavy chain for simplicity even though various positions in the both chains were tested in this study.

In this study, we report a method to generate site-specific conjugates of IgG and PE24. Trastuzumab, a human epidermal growth factor receptor 2 (Her2)-targeting IgG, was engineered with an unpaired Cys, and an unnatural amino acid having the azido group was incorporated into PE24 to install the bioorthogonal reactive moiety at the toxin protein. The two proteins were conjugated via a linker having bifunctional groups of maleimide and dibenzocyclooctyne (DBCO), which are reactive with the thiol and azido groups, respectively (Scheme 1).

## Results and discussion

### Introduction of the azido group into PE24 using an unnatural amino acid mutagenesis method

**Fig. 1:**
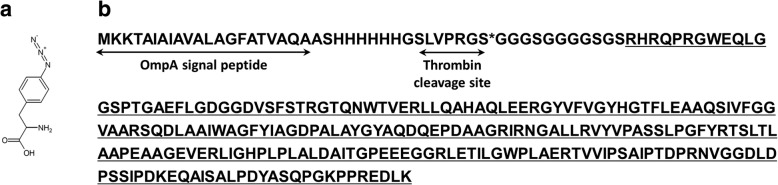
(**a**) Chemical structure of azidophenylalanine (AzF). **b** Amino acid sequence of the PE24-TAG construct. The amber codon (TAG) is indicated by * and the amino acid sequence of PE24 is underlined

The PE24 domain including the azido group was prepared by incorporating azidophenylalanine (AzF; Fig. 1a) in response to the amber (TAG) codon using an engineered orthogonal pair of tRNA_CUA_ and tyrosyl-tRNA synthetase derived from *Methanococcus jannaschii* [41, 42]. The C-terminal sequence of PE24 is important in its translocation into the cytoplasm by directing the toxin into the endoplasmic reticulum after endocytosis [43]. Thus, the reactive moiety was installed in the N-terminus with flexible linkers (Fig. 1b). The recombinant protein was purified using a nickel-immobilized resin from the periplasmic fraction, and the purification tag of His_6_ was removed by a thrombin reaction. The resulting protein was designated PE24-AzF.

### Determination of Cys position in trastuzumab for introduction of the thiol group

The method based on a substituted Cys residue was used for the site-specific conjugation of trastuzumab to PE24-AzF. At first, we decided to test a mutation of the heavy chain (HC)-A114C, which is one of two mutations that was used to develop an antibody-drug conjugate for the first time [44]. We constructed this variant and prepared antibody using the HEK293F mammalian expression system. The introduced Cys residues are usually modified during expression [29, 44]. The trastuzumab variants were reduced and reoxidized to generate the thiol group at position HC-A114 position as previously described [44] using 10-fold Tris(2-carboxyethyl) phosphine (TCEP) for reduction and 20-fold dehydroascorbic acid (dhAA) for reoxidation. Reactive Cys residues present in trastuzumab-HC-A114C were quantified using 4,4′-dithiodipyridine (4-PDS) [45]. The thiol-to-antibody ratio was 0.27 for the reoxidized trastuzumab-HC-A114C. This ratio was much lower than the theoretical value of 2 (see Additional file 1a). We hypothesized that the reduction potential was not sufficient to regenerate the thiol group at HC-A114C position, but an increase of TCEP concentration (20-fold) resulted in only a small increase in the value (0.76 thiol per antibody) (see Additional file 1a). However, reduction with 100-fold TCEP impaired the reoxidation efficiency. The band sizes were smaller than that of the fully assembled IgG (see Additional file 1b, lane 1), which implies that interchain disulfide bonds were not formed. Next, we constructed the other variant having the light chain (LC)-V205C mutation [44]. Trastuzumab-LC-V205C exhibited an even lower reoxidation yield (see Additional file 1b, lane 2) than trastuzumab-HC-A114C.

**Fig. 2:**
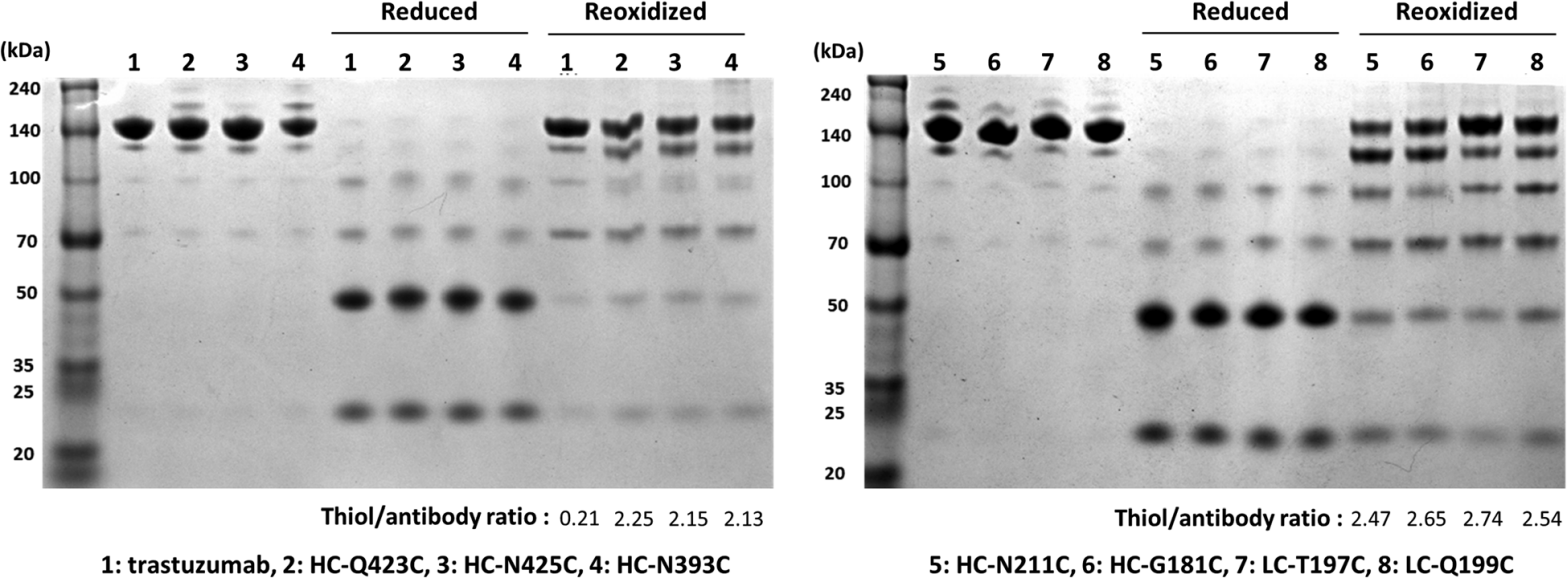
Reduction and reoxidation of trastuzumab variants. Reduced or reoxidized IgGs were analyzed by SDS-PAGE under a non-reducing condition. The trastuzumab variants were reduced with Tris(2-carboxyethyl) phosphine (TCEP) and then reoxidized in the presence of dehydroascorbic acid (dhAA). The thiol/antibody ratios of reoxidized IgGs were determined using 4,4′-dithiodipyridine (4-PDS), and the values are shown under the images

The unexpected results with the two trastuzumab variants prompted us to consider a recently published study [32] in which positions for Cys in IgG were systematically evaluated in a high-throughput method. Seven mutations (HC-Q423C, HC-N425C, HC-N393C, HC-N211C, HC-G181C, LC-T197C, and LC-Q199C) were chosen based on the drug-to-antibody ratio (DAR), and trastuzumab variants having each mutation were constructed. All trastuzumab antibodies were reduced with 100-fold TCEP and then oxidized with 20-fold dhAA. While the reoxidized trastuzumab showed 0.21 thiol per antibody, all the variants had a ratio between 2 and 3. The results of sodium dodecyl sulfate polyacrylamide gel electrophoresis (SDS-PAGE) and the thiol-to-antibody ratios (Fig. 2) indicated the incomplete formation of interchain disulfide bonds. The degree of bond formation was dependent on the positions for Cys. In particular, HC-N211C and HC-G181C displayed a markedly lower efficiency of reoxidation and thus were not evaluated for conjugation with PE24-AzF. These results suggested that the reoxidation efficiency of interchain disulfide bonds is affected by the position at which Cys is introduced. To our knowledge, this phenomenon has not been systematically evaluated and deserves further consideration.

**Fig. 3:**
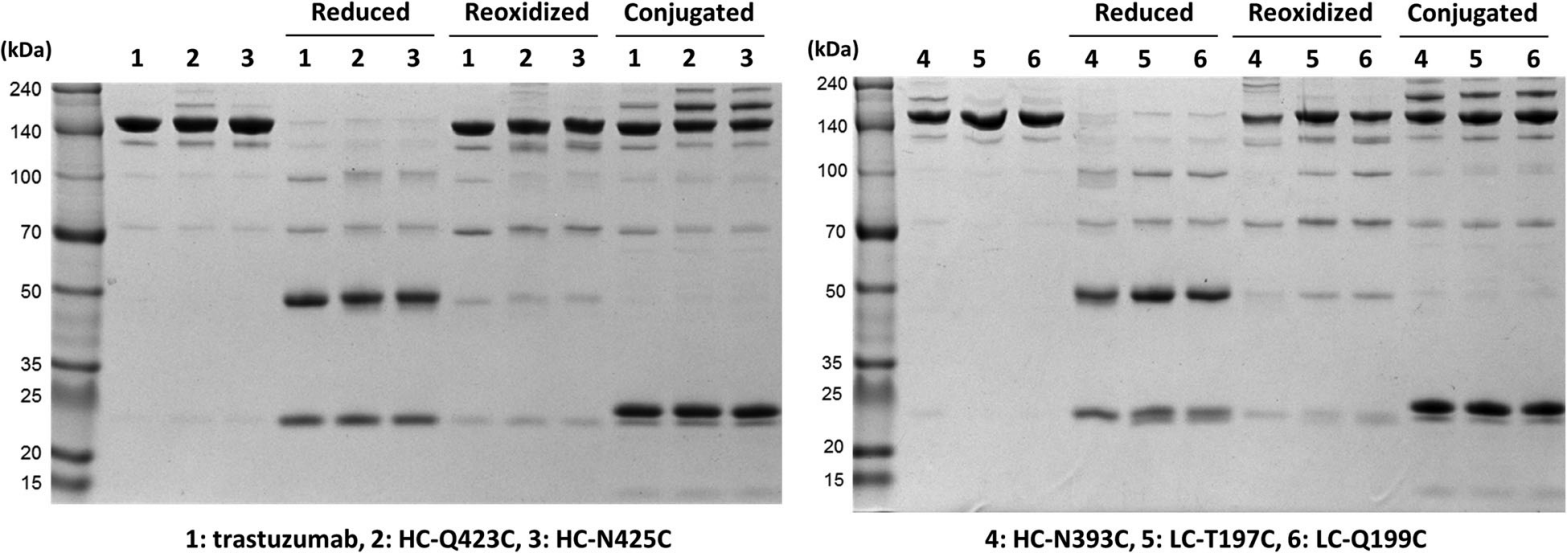
Conjugation of trastuzumab variants and PE24-AzF. Analysis of SDS-PAGE was done under a non-reducing condition for reduced, reoxidized, and PE24-conjugated trastuzumab. The reduced/reoxidized trastuzumab were first conjugated to DBCO-polyethylene glycol (PEG_4_)-maleimide using the thiol-maleimide coupling reaction. The modified antibodies were then reacted with PE24-AzF via the strain-promoted azide-alkyne cycloaddition reaction

We next evaluated the conjugation efficiency of PE24-AzF with HC-Q423C, HC-N425C, HC-N393C, LC-T197C, or LC-Q199C trastuzumab variants (Scheme 1). The reduced/reoxidized trastuzumab variants were first conjugated to DBCO-polyethylene glycol (PEG_4_)-maleimide using the thiol-maleimide coupling reaction. The modified antibodies were then reacted with PE24-AzF via the strain-promoted azide-alkyne cycloaddition reaction [46, 47]. The HC-Q423C and HC-N425C variants displayed higher conjugation yields than the other variants (Fig. 3). We decided to use trastuzumab-HC-N425C for further study because of its higher expression yield (see Additional file 2).

### Preparation of trastuzumab-HC-N425C and PE24-AzF conjugate

**Fig. 4:**
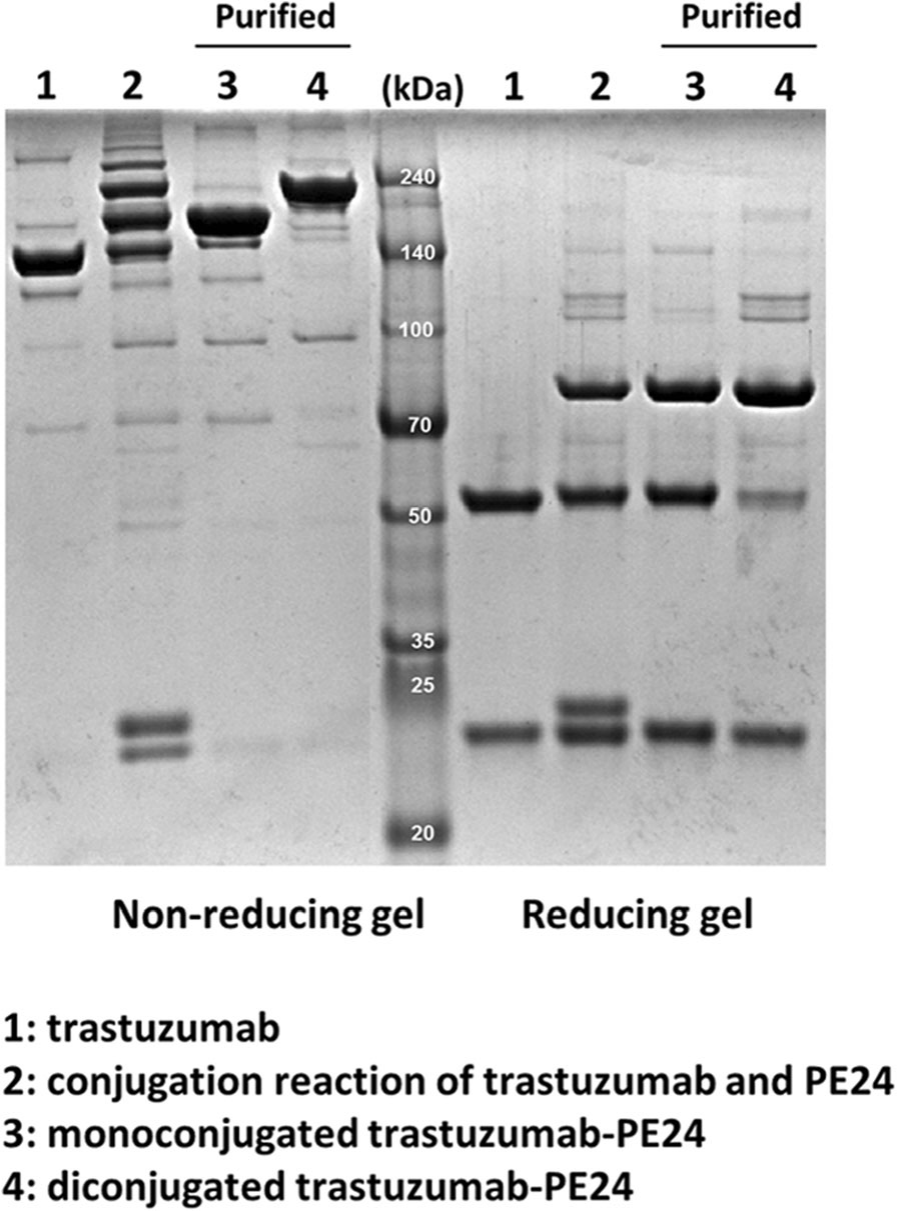
Purification of the trastuzumab-PE24 conjugate. The mono-conjugated trastuzumab-PE24 conjugate was purified by two-step purification using size exclusion and anion exchange chromatography. The full image of Fig. 4 is shown in Additional file 3b

Trastuzumab-HC-N425C was conjugated to PE24-AzF following the method described above with the slight modification of an extended time (from 4 to 16 h) for the reaction between the two protein molecules to improve the yield. A two-step purification method consisting of size exclusion and anion exchange chromatography was used to isolate trastuzumab-PE24 conjugates. Unconjugated PE24 and high-molecular weight aggregates were removed by size exclusion chromatography, and trastuzumab-PE24 was separated according to the number of conjugated PEs using the anion exchange chromatography (see Additional file 3a and b). We decided to use the mono-conjugated form for further characterizations for two reasons. First, the mono-conjugated form exhibited a slightly higher yield than the di-conjugated form. Second, one molecule of toxin per antibody could be sufficient to develop an immunotoxin due to the high cytotoxicity of PE. However, we expected that increasing the ratio of PE to IgG for conjugation reaction could result in a higher yield for the di-conjugated form. The result of SDS-PAGE for the mono-conjugated form under the reducing condition revealed that only the HC band was shifted as much as the molecular weight of PE24 (Lane 3 and 4 under the reducing condition of Fig. 4), which suggested that PE24-AzF was conjugated to the HC-N425C position in a site-specific manner.

### Functions of toxin and IgG of trastuzumab-PE24

**Fig. 5:**
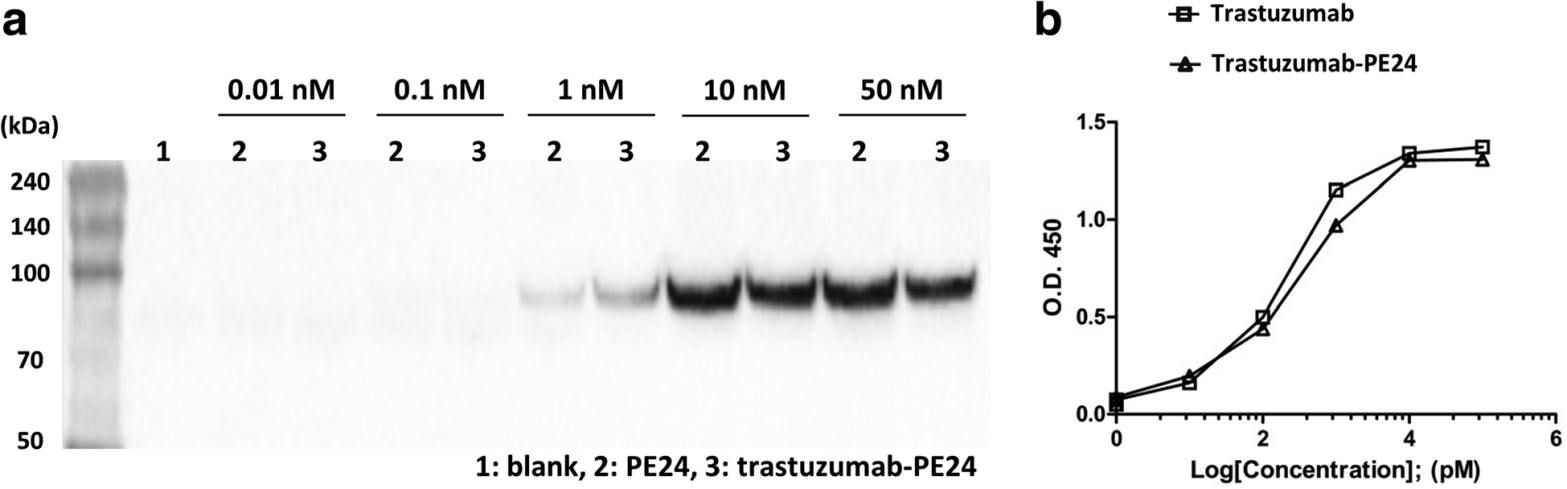
(**a**) ADP-ribosylation of eEF-2. Wheat germ extract including eEF-2 was incubated with biotinylated NAD^+^ in the presence of PE24-AzF or trastuzumab-PE24, and then the reactions were analyzed by western blot using streptavidin-HRP conjugate. **b** Binding analysis of trastuzumab and trastuzumab-PE24 on Her2 antigen by ELISA. The analyses were done three times. The error bars represent one standard deviation

**Fig. 6:**
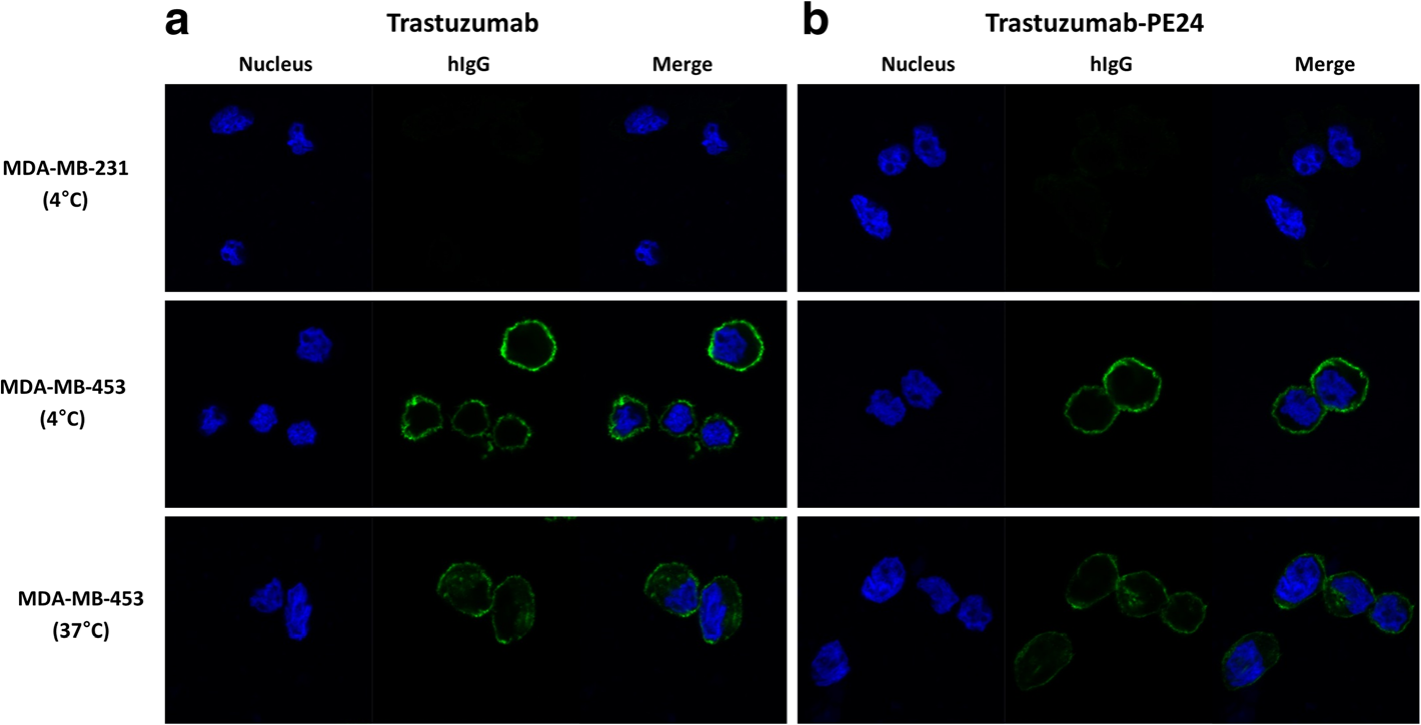
Binding and uptake of trastuzumab (**a**) and trastuzumab-PE24 (**b**) by Her2-positive cells. MDA-MB-231 (Her2-negative) or MDA-MB-453 (Her2-positive) cells were incubated with trastuzumab or trastuzumab-PE24 for 30 min at 4 °C. For inducing the cellular endocytosis pathways, the MDM−/MB-231 cells were further incubated at 37 °C for 4 h. The cells were stained with anti-human IgG antibody-Alexa 488 and Hoechst, and the images were obtained using confocal fluorescence microscopy

**Fig. 7:**
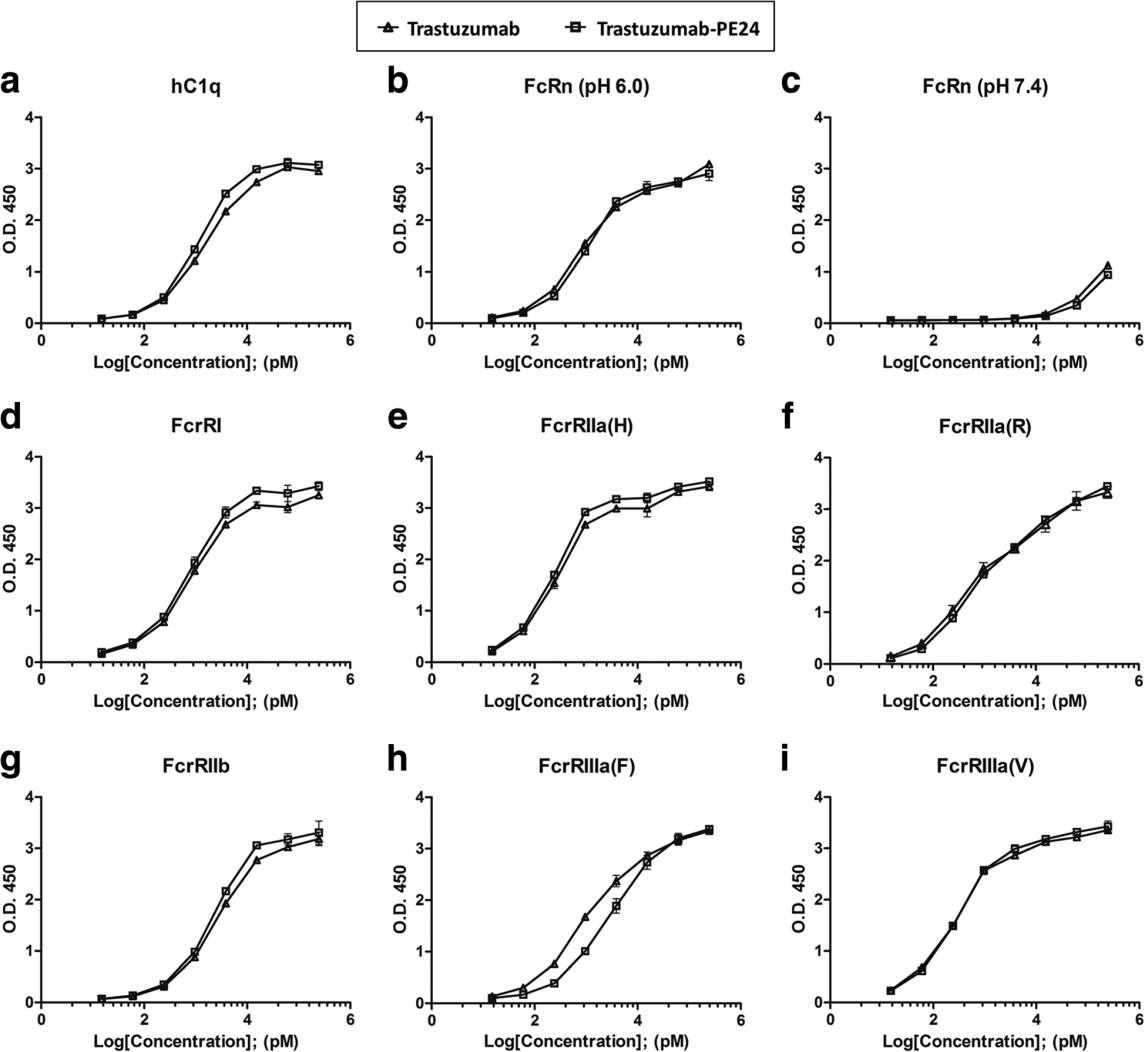
Binding of the trastuzumab-PE24 conjugate to Fc-receptors measured by ELISA: (**a**) hC1q, (**b**) FcRn at pH 6.0, (**c**) FcRn at pH 7.4, (**d**) FcrRI, (**e**) FcrRIIa(H), (**f**) FcrRIIa(R), (**g**) FcrRIIb, (**h**) FcrRIIIa(F), (**i**) FcrRIIIa(V). hC1q: complement component 1q; FcRn: neonatal Fc receptor; FcrRI: Fc gamma receptor I; FcrRIIa(H): Fc gamma receptor IIa (H134); FcrRIIa(R): Fc gamma receptor IIa (R134); FcrRIIb: Fc gamma receptor IIb; FcrRIIIa(F): Fc gamma receptor IIIa (F158); FcrRIIIa(V): Fc gamma receptor IIIa (V158). The analyses were done three times, and the error bars represent one standard deviation

Chemical modification of proteins can affect their original functions, and trastuzumab-PE24 was characterized concerning the functions of the toxin and IgG. PE24 induces cytotoxicity by ADP-ribosylation of eEF-2, and the enzymatic activity was tested for PE24-AzF and trastuzumab-PE24 [48]. The eEF-2 in wheat germ extract was incubated with biotinylated NAD^+^ in the presence of PE24-AzF or trastuzumab-PE24, and the reaction mixtures were analyzed by western blotting using streptavidin-horseradish peroxidase (HRP). The trastuzumab-PE24 conjugate displayed activity comparable to PE24-AzF (Fig. 5a). There was no difference in the Her2 binding affinity between trastuzumab and trastuzumab-PE24 as measured by enzyme-linked immunosorbent assay (ELISA) (Fig. 5b and Additional file 4). In addition, the conjugation of PE24 to trastuzumab did not affect its receptor-mediated endocytosis (Fig. 6), which is the first step for a PE-based immunotoxin to act on target cells. The trastuzumab-PE24 conjugate bound to only Her2-positive MDA-MB-453 cells the same as trastuzumab (second row of Fig. 6). The additional incubation of the cells at 37 °C resulted in the internalization of both trastuzumab and trastuzumab-PE24 (third row of Fig. 6). The conjugate interacted with various Fc receptors similar to trastuzumab (Fig. 7 and Additional file 4). These interactions play important roles in regulating IgG concentrations in serum and immune responses against pathogens, and thus could provide additional biological functions to immunotoxins based on full-length IgGs compared to recombinant immunotoxins with antibody fragments. The conjugation position of HC-N425C was located close to the C-terminus of the heavy chain (see Additional file 5) distant from the interaction positions with antigen and Fc receptors. These results suggested that the site-specific modification and careful selection of conjugation positions used to conjugate IgG and PE24 does not interfere with their original functions.

### Cytotoxicity of trastuzumab-PE24 against Her2 positive cell lines

**Fig. 8:**
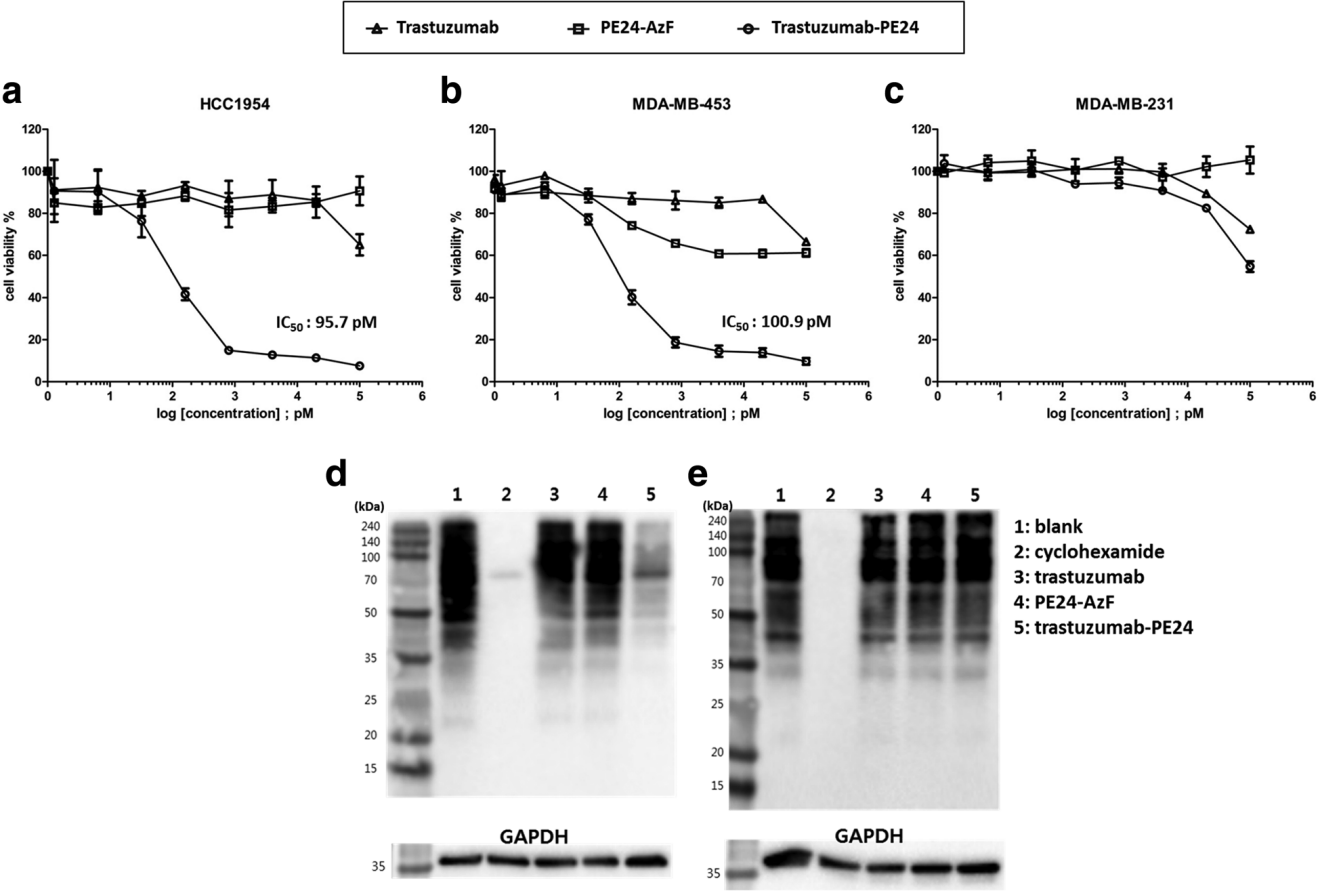
**a-c** Cytotoxicity of trastuzumab-PE24 on (a) HCC1954 (Her2 positive), (**b**) MDA-MB-453 (Her2 positive), and (**c**) MDA-MB-231 (Her2 negative). Cell viability was measured by the WST-8 assay. The IC_50_ of trastuzumab-PE24 for HCC1954 and MDA-MB-453 was 95.7 pM and 100.9 pM, respectively. The analyses were performed three times. The error bars represent one standard deviation. **d-e** Inhibition of protein synthesis by trastuzumab-PE24 in (**d**) HCC1954 cells and (**e**) MDA-MB-231 cells. Cellular proteins were labeled with a methionine surrogate of azidohomoalanine (AHA) after treating with trastuzumab, PE24, or trastuzumab-PE24. The presence of azide of AHA in proteome was detected by western blot using streptavidin-HRP conjugate after the Cu(I)-catalyzed click reaction with biotin-PEG_4_-alkyne. GAPDH: glyceraldehyde-3-phosphate dehydrogenase

The cytotoxicity of trastuzumab-PE24 was evaluated for the HCC1954 and MDA-MB-453 Her2-positive cell lines and a Her2-negative cell line of MDA-MB-231. The immunotoxin efficiently induced cell death for the Her2-positive cell lines, with minimal cytotoxicity toward the Her2-negative cell line (Fig. 8a-c). The latter activity was comparable to that of the unconjugated PE24. The results indicated the specificity of the trastuzumab-PE24 conjugate for Her2-overexpressing cells. Of note, HCC1954 and MDA-MB-453 cells are resistant to trastuzumab [49, 50]. Inhibition of protein synthesis by trastuzumab-PE24 was demonstrated using the bioorthogonal noncanonical amino acid tagging (BONCAT) method [51]. BONCAT relies on the incorporation of bio-orthogonally reactive amino acid analogs into the proteome. For example, azidohomoalanine (AHA) that has an azido group can be incorporated into methionine positions, especially when the concentration of methionine is low. The reactive group can be modified with an alkyne-containing probe, such as biotin-alkyne, via the Cu(I)-catalyzed click reaction [52, 53]. The lack of the probe in proteins indicates the inhibition of protein synthesis. Only trastuzumab-PE24 treatment for the Her2-positive cell resulted in the decrease of the western blot signal detected by streptavidin-HRP (Fig. 8d and e). The results indicated that the trastuzumab-PE24 conjugate induces the inhibition of protein synthesis specifically in Her2-positive cells, which is one of the main causes of the induced cytotoxicity.

## Conclusions

In this study, we developed a PE-based immunotoxin using IgG via a site-specific conjugation method using an engineered Cys residue in the antibody and a bio-orthogonally reactive unnatural amino acid at the toxin. AzF containing the azido group was incorporated into an engineered PE (PE24), resulting in PE24-AzF. Position HC-N425 of trastuzumab was selected for the introduction of Cys based on thiol reactivity, reoxidation efficiency of interchain disulfide bonds, and conjugation efficiency with PE-AzF. The conjugation reaction took place at the position where Cys was introduced, and a mono-conjugated immunotoxin was purified using a two-step chromatography process. The immunotoxin was markedly cytotoxic for Her2 positive cells via the inhibition of protein synthesis. The results presented in this study clearly show that the conjugation method does not interfere with the functions of IgG. It has also been reported that regulatory T-cell epitopes present in the Fc domain participate in suppressing the generation of anti-drug antibody against IgG [54, 55]. The generation of an anti-drug antibody against PE24 was recently reported [56], which suggests that an immunotoxin based on IgG might be able to address the challenges concerning immunogenicity. Studies are planned to evaluate the trastuzumab-PE24 conjugate for tumor regression, half-life in blood, and in vivo immunogenicity using animal models. In addition, considering the minimal modifications of IgG and protein for conjugation, the method described in this study can be used to develop other antibody-protein conjugates for applications in therapeutics and diagnostics. In particular, we expect that other PE-based immunotoxins can be easily constructed by using IgGs approved or in development; many of which are the same Fc isotype of IgG1 as trastuzumab.

## Methods

### Construction of plasmids

A cysteine (Cys) residue was introduced into each position of trastuzumab by assembly PCR (see Additional file 6) using the plasmid including the trastuzumab gene [57] as the template. The PCR product and the pcDNA 3.1 plasmid were digested with NotI and XhoI (New England Biolabs). The digested DNAs were ligated with T4 DNA ligase (New England Biolabs) and the product was transformed into *Escherichia coli* DH10β. A gene for PE24-TAG (Fig. 1a) synthesized by Bioneer (Korea) was cloned into the pET21a plasmid using NdeI and NotI (New England Biolabs), resulting in pET21a-PE24-TAG.

### Expression and purification of trastuzumab variants

For transfection with 200 ml culture, 250 μg of plasmids expressing heavy and light chain of trastuzumab and 750 μg polyethyleneimine (PEI, Polyscience) were each added to 5 ml of Freestyle medium (Gibco). After incubation for 10 min at room temperature, the plasmids were filtered and added to the PEI solution. The mixture was incubated for 10 min at room temperature, and then transferred to a 100-ml suspension of HEK293F cells (2 × 10^6^ cells/ml; Invitrogen). The culture was incubated for 4 h at 37 °C in the presence of 8% CO_2_ with shaking at 120 rpm. Ninety milliliters of medium was added to the culture and incubated for 7 days. The culture was harvested by centrifugation (3000×g, 4 °C, 15 min). The supernatant was filtered through a bottle-top filter (Corning) and bound to Protein A resin (Captiva) for 1 h at 4 °C. The resin was washed with phosphate-buffered saline (PBS, pH 7.4 here and hereafter) and trastuzumab was eluted with 0.1 M glycine (pH 3.0). A 1/40 volume of 1 M Tris (pH 9.0) was added to the eluted trastuzumab to neutralize the preparation. The buffer was exchanged with PBS by centrifugal filtration using a molecular weight cutoff (MWCO) of 100 kDa (Millipore).

### Expression and purification of PE24-AzF

Three plasmids, pET21a-PE24-TAG, pEVOL [58] containing the tRNA/azidophenylalanyl-tRNA synthetase (AzF-RS) pair originated from *Methanococcus jannaschii* [41], and pBbS2K containing *E. coli* prolyl-tRNA synthetase (ProRS) [59, 60] were co-transformed in *E. coli* BL21(DE3). The cells were cultured in 400 ml 2 × YT medium at 37 °C. The expression of AzF-RS and ProRS was induced by adding 0.2% L-arabinose (ROTH) and 50 nM anhydrotetracycline (Sigma-Aldrich) when the optical density at 600 nm (OD_600_) reached 0.5. The expression of PE24 and incorporation of AzF were induced by adding 1 mM isopropyl β-D-1-thiogalactopyranoside (Bioshop) and 2 mM azidophenylalanine (AzF, Bachem) at an OD_600_ of 1.0. The cells were cultured at 25 °C overnight and harvested by centrifugation (9300×g, 4 °C, 15 min). The cell pellet obtained from each 400-ml culture was resuspended in 11 ml of 0.75 M sucrose / 0.1 M Tris (pH 8.0) and 1.5 mg lysozyme was added. Twenty-one milliliters of 1 mM EDTA was added and incubated on ice for 10 min with mixing by inversion. MgCl_2_ (0.5 M, 1.5 ml) was added to the mixture and incubated on ice for 10 min with mixing by inversion. The supernatant was collected by centrifugation (9300×g, 4 °C, 15 min) and incubated with Ni-NTA resin (Clontech) at 4 °C for 1 h. The Ni-NTA resin was washed with wash buffer (50 mM NaH_2_PO_4_, 300 mM NaCl, 40 mM imidazole; pH 7.4). PE24-AzF was eluted with elution buffer (50 mM NaH_2_PO_4_, 300 mM NaCl, 300 mM imidazole; pH 7.4). The His_6_ tag was removed from the purified PE24-AzF by treatment with thrombin (GE Healthcare).

### Reduction and reoxidation of trastuzumab variants

The solution of IgGs (4 mg/ml) was incubated with 100-fold TCEP (Sigma-Aldrich) in reduction buffer (20 mM Tris-HCl, 1 mM EDTA, pH 7.4) at 37 °C for 3 h. The protein solution was exchanged with a reoxidation buffer (50 mM Tris-HCl, 150 mM NaCl, pH 7.5) using the centrifugal filter with a 100 kDa MWCO. The reduced IgGs (1 mg/ml) were reoxidized with 20-fold dehydroascorbic acid (Sigma-Aldrich) in the reoxidation buffer at 25 °C for 3 h, and the solutions were exchanged with PBS using the aforementioned centrifugal filter.

### Conjugation of trastuzumab variants and PE24-AzF

The reoxidized IgGs were incubated with a 40-fold molar excess maleimide-(PEG)_4_-DBCO linker (Click Chemistry Tools) in PBS at 25 °C for 2 h. The unreacted linker was removed by buffer exchange using the centrifugal filter with a 100 kDa MWCO. The resulting IgGs were conjugated with 4-fold molar excess PE24-AzF at 4 °C.

### Purification of trastuzumab-PE24 conjugate

The trastuzumab-PE24 conjugate was purified by size exclusion chromatography and anion exchange chromatography using the Superdex 200 column and Mono-Q column, respectively (both from GE Healthcare). The Superdex 200 column was equilibrated with running buffer (10 mM phosphate, 1 M NaCl, pH 7.4). The trastuzumab-PE24 conjugate was injected into this column and eluted with the running buffer to remove the unreacted PE24-AzF. The eluted fractions of the trastuzumab-PE24 conjugate and unconjugated trastuzumab were diluted with 50 ml of dilution buffer (10 mM phosphate, pH 7.4) and loaded onto a Mono-Q column equilibrated with buffer A (20 mM phosphate, pH 7.0). Trastuzumab-PE24 was eluted with a gradient of buffer A and buffer B (20 mM phosphate, 1 M NaCl, pH 7.0); 10–15% B for trastuzumab conjugated one PE24 molecule, 15–20% B for trastuzumab conjugated two PE24 molecules, and 20–25% B for trastuzumab conjugated three PE24 molecules.

### Enzyme-linked immunosorbent assay

For the antigen binding assay, ErBb2 (Her2; Sino Biological) dissolved in PBS including 0.05% sodium azide was used to coat wells of a 96-well plate by incubation at 4 °C for 16 h. The wells were washed three times with 25 mM Tris, 150 mM NaCl, 0.05% Tween 20, pH 7.5 (TBST), and then were blocked with PBS including 20 mg/ml bovine serum albumin (PBSB) at 25 °C for 2 h. Trastuzumab or trastuzumab-PE24 was added to each well and incubated at 25 °C for 1 h. After washing the wells three times with TBST, protein L-horseradish peroxidase (HRP) conjugate (Thermo Fisher Scientific) was added to each well and incubated at 25 °C for 1 h After washing the wells three times with TBST, 3,3′,5,5′-tetramethylbenzidine (TMB) substrate (Thermo Fisher Scientific) was added to each well and the absorbance was measured at 450 nm. For Fc-related receptor binding assay, 4 μg/ml of trastuzumab or trastuzumab-PE24 in 0.05 M Na_2_CO_3_ pH 9.6 was used to coat wells of a 96-well plate at 4 °C overnight. The coated wells were blocked with PBSB at 25 °C for 2 h. The wells were washed three times with PBS including 0.05% Tween 20 (PBST). Fifty microliters of the C1q and Fc receptors serially diluted in PBSB were added to the wells and incubated at 25 °C for 1 h. The C1q and FcrRI-His_6_ were purchased from Abcam and R&D Systems, respectively. FcRn-GST, FcrIIa(H)-GST, FcrIIa(V)-GST, FcrIIb-GST, FcrIIIa(F)-GST, and FcrIIIa(V)-GST were prepared as previously described [61]. After the wells were washed three times with PBST, anti-His-HRP conjugate (Sigma-Aldrich) was added to the wells for FcrRI-His_6_, anti-GST-HRP conjugate (GE Healthcare) for FcRn-GST, FcrIIa(H)-GST, FcrIIa(V)-GST, FcrIIb-GST, FcrIIIa(F)-GST, and FcrIIIa(V)-GST, and anti-C1q-HRP conjugate (Sigma-Aldrich) for C1q. The plates were incubated at 25 °C for 1 h. After washing the wells three times with PBST, TMB substrate was added to each well and the absorbance was measured at 450 nm.

### ADP-ribosylation assay of eEF-2

Trastuzumab-PE24 conjugate or PE24-AzF (1 nM) was incubated with wheat germ extract (Promega) and 50 nM biotinylated NAD^+^ (CPC Scientific) in 20 mM Tris-HCl buffer (pH 7.4) containing 1 mM EDTA and 1 mM dithiothreitol at 37 °C for 1 h [24, 62]. The reaction was quenched by PAGE sample buffer containing SDS. The biotinylated eEF-2 was detected by western blotting using a streptavidin-HRP conjugate (Thermo Fisher Scientific). Western blot images were analyzed using the ChemiDoc XRS system (Bio-Rad Laboratories).

### Immunostaining and confocal fluorescence microscopy

MDA-MB-231 or MDM-MB-453 cells (4 × 10^4^) were seeded in RPMI 1640 medium including 10% FBS and 1% penicillin/streptomycin on coverslips (Marienfeld) in a 24-well plate and grown at 37 °C for 24 h in an atmosphere of 5% CO_2_. The cells were treated with 200 nM trastuzumab or trastuzumab-PE24 at 4 °C for 30 min to allow binding to the cells or were followed by an additional incubation at 37 °C for 4 h to induce the cellular endocytosis pathways. The cells were washed with PBS and fixed with 4% *p*-formaldehyde for 10 min, washed with PBS, and permeabilized with PBS containing 0.1% (w/v) saponin, 0.1% (w/v) sodium azide, and 1% (w/v) bovine serum albumin (BSA) for 10 min. The cells were washed with PBS and blocked with 1% (w/v) BSA in PBS for 1 h, followed by exposure to anti-human IgG antibody-Alexa 488 (Thermo Fisher Scientific) for 1 h. Cell nuclei were stained with Hoechst (Thermo Fisher Scientific) for 10 min. Incubation and staining were performed at room temperature. The stained cells were examined by confocal microscopy using a LSM710 confocal microscope equipped with a 63× objective (Carl Zeiss). The images were analyzed using ZEN software (Carl Zeiss).

### Protein synthesis inhibition assay

Inhibition of protein synthesis of cells by trastuzumab-PE24 was evaluated by the BONCAT method [51]. Her2-positive BT-474 cells and MDA-MB-231 Her2-negative cells were seeded (5 × 10^4^) with RPMI 1640 medium (HyClone) containing 10% FBS (HyClone) and 1% penicillin/streptomycin (HyClone) in 6-well plates and incubated at 37 °C for 24 h in an atmosphere of 5% CO_2_. Trastuzumab, PE24, or trastuzumab-PE24 (0.1 nM) was added to the wells and incubated at 37 °C for 20 h. Azidohomoalanine (Click Chemistry Tools) (4 mM) was added to each well and incubated at 37 °C for 2 h. The cells were washed with chilled PBS including 1 mM MgCl_2_ and 0.1 mM CaCl_2_. The washed cells were trypsinized and harvested. The harvested cells were lysed with 1% (w/v) SDS, and the cell lysate was reacted with biotin-PEG_4_-alkyne (Click Chemistry Tools) by the Cu(I)-catalyzed click reaction. The biotinylated proteins were detected by western blotting using the streptavidin-HRP conjugate.

### Cell viability assay

Cells (2 × 10^3^) were seeded in RPMI 1640 medium (HyClone) including 10% FBS and 1% penicillin/streptomycin in wells of a 96-well plate and grown at 37 °C for 24 h in an atmosphere of 5% CO_2_. Trastuzumab, PE24-AzF, or trastuzumab-PE24 was added to the wells and incubated for 72 h. Ten microliters of WST-8 reagent (Dojindo) was added to each well and incubated for 1 h, and the absorbance was measured at 450 nm.

## Additional files

Additional file 1: Reduction and reoxidation of trastuzumab and trastuzumab variants. (a) Reoxidation of trastuzumab-HC-A114C reduced with different concentrations of TCEP. Lane 1: Trastuzumab-HC-A114C; Lane 2: Reduction with 10-fold TCEP; Lane 3: Reduction with 20-fold TCEP. (b) Reoxidation of trastuzumab reduced with 100-fold TCEP. Lane 1: Trastuzumab-HC-A114C; Lane 2: Trastuzumab-LC-V205C. SDS-PAGE analyses were done under a non-reducing condition. The thiol/antibody ratios are presented under the SDS-PAGE images. (PDF 98 kb)

Additional file 2: Production yields of trastuzumab variants. (PDF 5 kb)

Additional file 3: Purification of trastuzumab-PE24 conjugate. (a) Purification by size exclusion chromatography. Each fraction was analyzed by SDS-PAGE, and fractions 21 to 25 were pooled for next purification. (b) Purification by anion exchange chromatography. The pooled fractions from the size exclusion chromatography were purified further by anion exchange chromatography. Fractions for each peak were pooled and then analyzed by SDS-PAGE. (PDF 112 kb)

Additional file 4: Apparent dissociation constants (K_d_s) of trastuzumab and trastuzumab-PE24 to the Her2 antigen and Fc receptors. The values were determined by fitting the results of Figs. 5b and 6 using PRISM software. The equation is *Y* = *B*_max_  × *X*/(K_d_ + *X*), where *Y* is the OD_450_ at each concentration, *X* is the concentration of antigen and receptors, and *B*_max_ is the OD_450_ at saturation. (PDF 118 kb)

Additional file 5: Positions of introduced cysteine residues for trastuzumab. The positions are indicated using a trastuzumab Fab structure (PDB: 1N8Z) (a) and a trastuzumab Fc structure (PDB: 3D6G) (b). Two positions (LC-T197C, LC-Q199C) are in the light chain (green) and five positions (HC-G181C, HC-N211C, HC-N393C, HC-Q423C, HC-N425C) are in the heavy chain (yellow). (PDF 141 kb)

Additional file 6: Primers used in this study. (PDF 70 kb)

## Data Availability

The datasets supporting the conclusion of this article are included within the article and the additional files.

## Abbreviations

4-PDS: 4,4′-Dithiodipyridine
AHA: Azidohomoalanine
AzF: Azidophenylalanine
BONCAT: Bioorthogonal noncanonical amino acid tagging
DAR: Drug-to-antibody ratio
DBCO: Dibenzocyclooctyne
dhAA: Dehydroascorbic acid
eEF-2: Eukaryotic elongation factor-2
IgG: Immunoglobulin
PE: *Pseudomonas* exotoxin A
PEG: Polyethylene glycol
TCEP: Tris(2-carboxyethyl)phosphine
