# Supplementary material for: Construction of an immunotoxin via site-specific conjugation of anti-Her2 IgG and engineered Pseudomonas exotoxin A
Source: J Biol Eng. 2019 Jun 21;13:56. doi: 10.1186/s13036-019-0188-x (PMC6588878; doi:10.1186/s13036-019-0188-x)
Supplement: Supplementary file 1 — Reduction and reoxidation of trastuzumab and trastuzumab variants. (a) Reoxidation of trastuzumab-HC-A114C reduced with different concentrations of TCEP. Lane 1: Trastuzumab-HC-A114C; Lane 2: Reduction with 10-fold TCEP; Lane 3: Reduction with 20-fold TCEP. (b) Reoxidation of trastuzumab reduced with 100-fold TCEP. Lane 1: Trastuzumab-HC-A114C; Lane 2: Trastuzumab-LC-V205C. SDS-PAGE analyses were done under a non-reducing condition. The thiol/antibody ratios are presented under the SDS-PAGE images. (PDF 98 kb) [file 13036_2019_188_MOESM1_ESM.pdf]

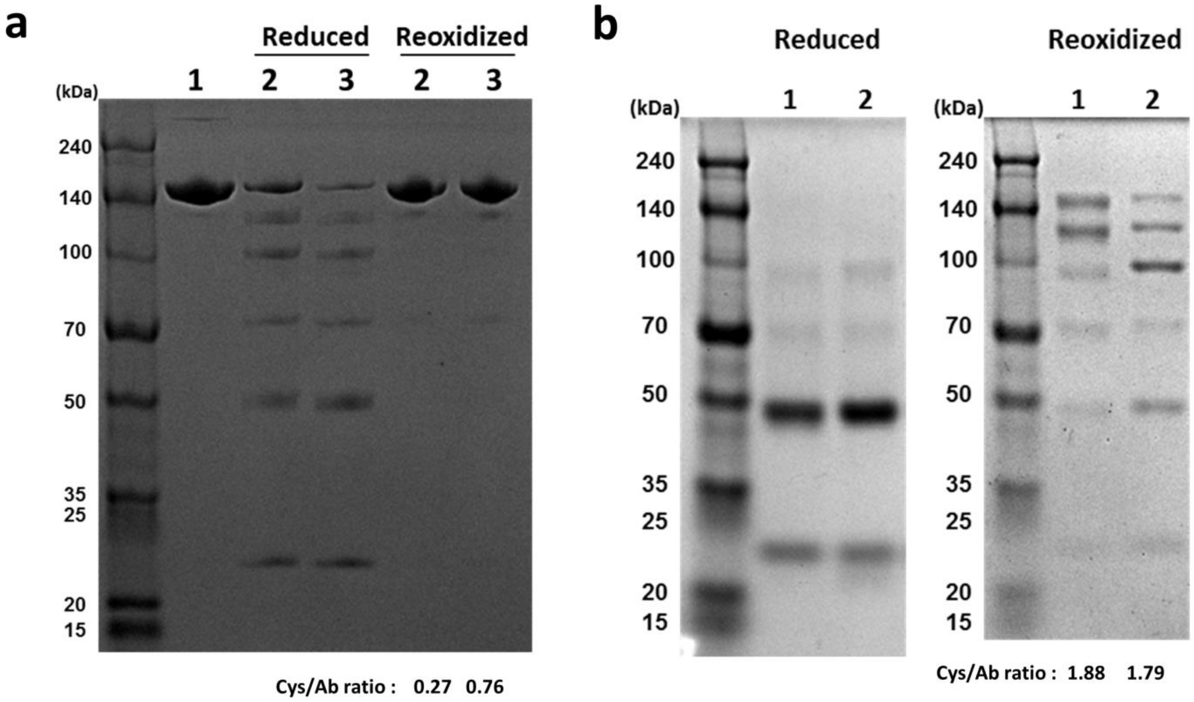

Additional file 1. Reduction and reoxidation of trastuzumab and trastuzumab variants. (a) Reoxidation of trastuzumab-HC-A114C reduced with different concentrations of TCEP. Lane 1: Trastuzumab-HC-A114C; Lane 2: Reduction with 10-fold TCEP; Lane 3: Reduction with 20-fold TCEP. (b) Reoxidation of trastuzumab reduced with 100-fold TCEP. Lane 1: Trastuzumab-HC-A114C; Lane 2: Trastuzumab-LC-V205C. SDS-PAGE analyses were done under a non-reducing condition. The thiol/antibody ratios are presented under the SDS-PAGE images.
