# Supplementary material for: Construction of an immunotoxin via site-specific conjugation of anti-Her2 IgG and engineered Pseudomonas exotoxin A
Source: J Biol Eng. 2019 Jun 21;13:56. doi: 10.1186/s13036-019-0188-x (PMC6588878; doi:10.1186/s13036-019-0188-x)
Supplement: Supplementary file 2 — Production yields of trastuzumab variants. (PDF 5 kb) [file 13036_2019_188_MOESM2_ESM.pdf]

Additional file 2. Production yields of trastuzumabs

	Trastuzumab	HC-Q423C	HC-N425C	HC-N393C	HC-N211C	HC-G181C	LC-T197C	LC-Q199C
Production yield (mg/L)	96	45	60	21	56	40	54	58
