# Supplementary material for: Construction of an immunotoxin via site-specific conjugation of anti-Her2 IgG and engineered Pseudomonas exotoxin A
Source: J Biol Eng. 2019 Jun 21;13:56. doi: 10.1186/s13036-019-0188-x (PMC6588878; doi:10.1186/s13036-019-0188-x)
Supplement: Supplementary file 3 — Purification of trastuzumab-PE24 conjugate. (a) Purification by size exclusion chromatography. Each fraction was analyzed by SDS-PAGE, and fractions 21 to 25 were pooled for next purification. (b) Purification by anion exchange chromatography. The pooled fractions from the size exclusion chromatography were purified further by anion exchange chromatography. Fractions for each peak were pooled and then analyzed by SDS-PAGE. (PDF 112 kb) [file 13036_2019_188_MOESM3_ESM.pdf]

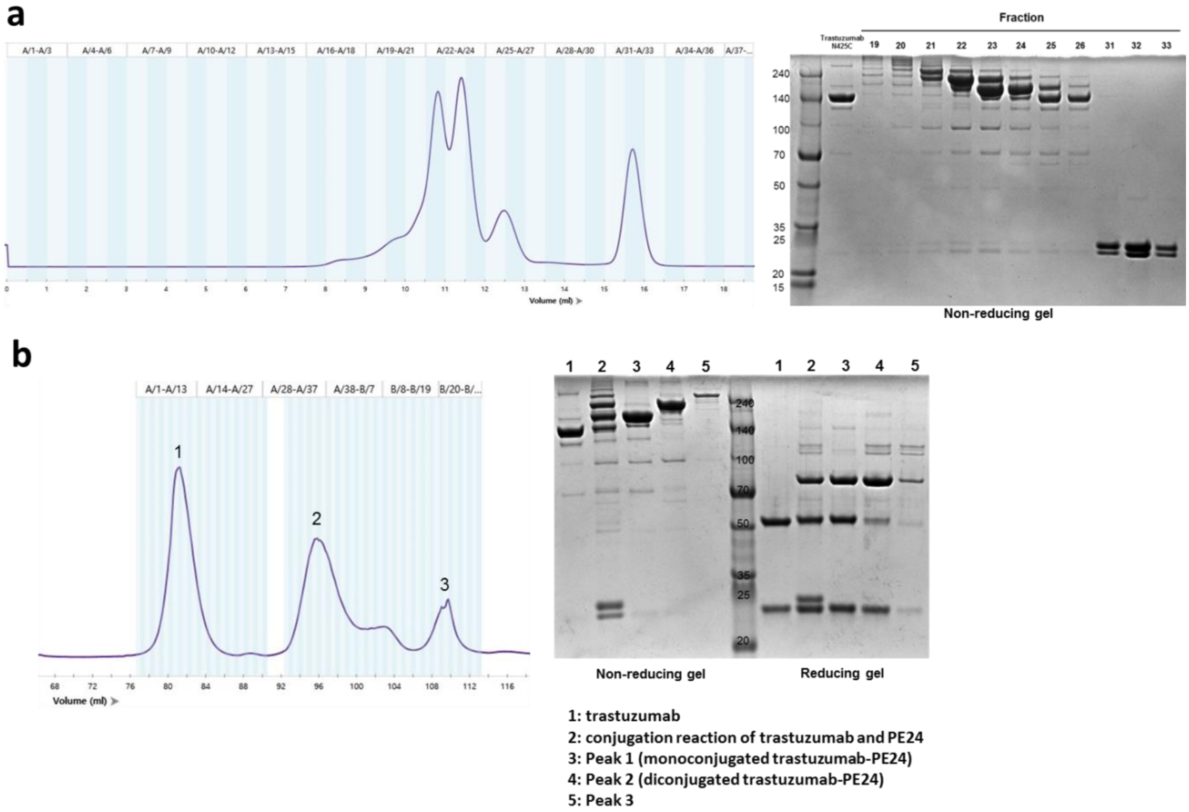

Additional file 3. Purification of trastuzumab-PE24 conjugate. (a) Purification by size exclusion chromatography. Each fraction was analyzed by SDS-PAGE, and fractions 21 to 25 were pooled for next purification. (b) Purification by anion exchange chromatography. The pooled fractions from the size exclusion chromatography were purified further by anion exchange chromatography. Fractions for each peak were pooled and then analyzed by SDS-PAGE.
