# Supplementary material for: Construction of an immunotoxin via site-specific conjugation of anti-Her2 IgG and engineered Pseudomonas exotoxin A
Source: J Biol Eng. 2019 Jun 21;13:56. doi: 10.1186/s13036-019-0188-x (PMC6588878; doi:10.1186/s13036-019-0188-x)
Supplement: Supplementary file 4 — Apparent dissociation constants (Kds) of trastuzumab and trastuzumab-PE24 to the Her2 antigen and Fc receptors. The values were determined by fitting the results of Figs. 5b and 6 using PRISM software. The equation is Y = Bmax × X/(Kd + X), where Y is the OD450 at each concentration, X is the concentration of antigen and receptors, and Bmax is the OD450 at saturation. (PDF 118 kb) [file 13036_2019_188_MOESM4_ESM.pdf]

| | $K_d$ (nM) | |
|---|---|---|
| | Trastuzumab | Trastuzumab-PE24 |
| Her-2 antigen | 0.165 ± 0.018 | 0.213 ± 0.041 |
| C1q | 1.445 ± 0.076 | 1.121 ± 0.055 |
| FcRn (pH 6.0) | 0.850 ± 0.095 | 0.974 ± 0.069 |
| FcRn (pH 7.4) | 171.4 ± 36.39 | 255.0 ± 85.33 |
| FcrRI | 0.722 ± 0.055 | 0.688 ± 0.057 |
| FcrRIIa(H) | 0.259 ± 0.029 | 0.227 ± 0.022 |
| FcrRIIa(R) | 0.630 ± 0.121 | 0.849 ± 0.134 |
| FcrRIIb | 2.482 ± 0.130 | 2.124 ± 0.149 |
| FcrRIIIa(F) | 0.913 ± 0.099 | 2.624 ± 0.235 |
| FcrRIIIa(V) | 0.266 ± 0.025 | 0.289 ± 0.024 |

Additional file 4. Apparent dissociation constants ($K_d$s) of trastuzumab and trastuzumab-PE24 to the Her2 antigen and Fc receptors. The values were determined by fitting the results of Figure 5b and 6 using PRISM software. The equation is $Y = B_{max} \times X/(K_d + X)$, where $Y$ is the $OD_{450}$ at each concentration, $X$ is the concentration of antigen and receptors, and $B_{max}$ is the $OD_{450}$ at saturation.
