# Supplementary material for: Construction of an immunotoxin via site-specific conjugation of anti-Her2 IgG and engineered Pseudomonas exotoxin A
Source: J Biol Eng. 2019 Jun 21;13:56. doi: 10.1186/s13036-019-0188-x (PMC6588878; doi:10.1186/s13036-019-0188-x)
Supplement: Supplementary file 5 — Positions of introduced cysteine residues for trastuzumab. The positions are indicated using a trastuzumab Fab structure (PDB: 1N8Z) (a) and a trastuzumab Fc structure (PDB: 3D6G) (b). Two positions (LC-T197C, LC-Q199C) are in the light chain (green) and five positions (HC-G181C, HC-N211C, HC-N393C, HC-Q423C, HC-N425C) are in the heavy chain (yellow). (PDF 141 kb) [file 13036_2019_188_MOESM5_ESM.pdf]

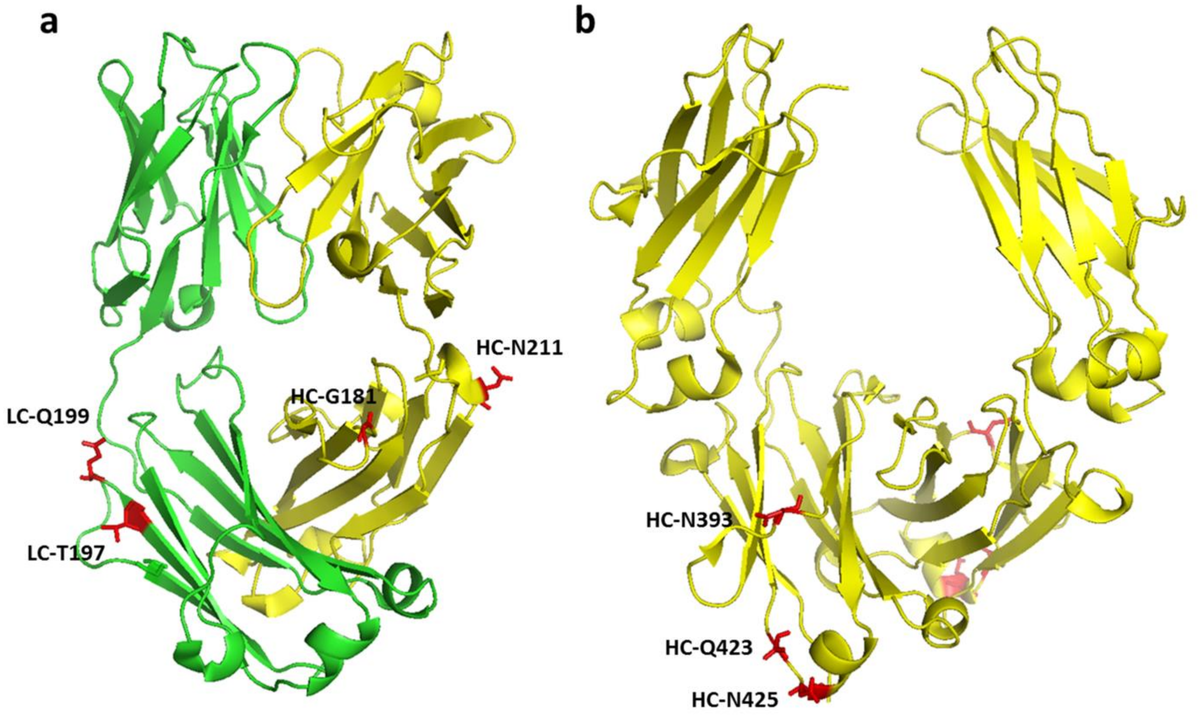

Additional file 5. Positions of introduced cysteine residues for trastuzumab. The positions are indicated using a trastuzumab Fab structure (PDB: 1N8Z(1)) (a) and a trastuzumab Fc structure (PDB: 3D6G(2)) (b). Two positions (LC-T197C, LC-Q199C) are in the light chain (green) and five positions (HC-G181C, HC-N211C, HC-N393C, HC-Q423C, HC-N425C) are in the heavy chain (yellow).

REFERENCES

1. Cho HS, Mason K, Ramyar KX, Stanley AM, Gabelli SB, Denney DW, Jr., et al. Structure of the extracellular region of HER2 alone and in complex with the Herceptin Fab. Nature. 2003 Feb 13;421(6924):756-60. PubMed PMID: 12610629.

2. Moiani D, Salvalaglio M, Cavallotti C, Bujacz A, Redzynia I, Bujacz G, et al. Structural characterization of a Protein A mimetic peptide dendrimer bound to human IgG. The journal of physical chemistry B. 2009 Dec 17;113(50):16268-75. PubMed PMID: 19924842.
