# Supplementary material for: Construction of an immunotoxin via site-specific conjugation of anti-Her2 IgG and engineered Pseudomonas exotoxin A
Source: J Biol Eng. 2019 Jun 21;13:56. doi: 10.1186/s13036-019-0188-x (PMC6588878; doi:10.1186/s13036-019-0188-x)
Supplement: Supplementary file 6 — Primers used in this study. (PDF 70 kb) [file 13036_2019_188_MOESM6_ESM.pdf]

Additional file 6. Primers used in this study

Primer		Sequence (5′→ 3′)
A	Trastuzumab-f	GATAGGCACCTATTGGTCTTACTGACATCC
B	Trastuzumab-r	GGGAAGCAATAGCATGATACAAAGG
1-f	HC-Q423C-f	AGCAGGTGGCAGTGCGGGAACGTCTTC
1-r	HC-Q423C-r	GAAGACGTTCCCGCACTGCCACCTGCT
2-f	HC-N425C-f	TGGCAGCAGGGGTGCGTCTTCTCATGC
2-r	HC-N425C-r	GCATGAGAAGACGCACCCCTGCTGCCA
3-f	HC-N393C-f	TGGGCAGCCGGAGTGCAACTACAAGACCA
3-r	HC-N393C-r	TGGTCTTGTAGTTGCACTCCGGCTGCCCA
4-f	HC-N211C-f	TCACAAGCCCAGCTGCACCAAGGTGGACA
4-r	HC-N211C-r	TGTCCACCTTGGTGCAGCTGGGCTTGTGA
5-f	HC-G181C-f	ACATGCCTCATGCCTCTACTCCC
5-r	HC-G181C-r	GGGAGTAGAGGCATGAGGACTGT
6-f	LC-T197C-f	GCCTGCGAAGTCTGCCATCAGGGCCTG
6-r	LC-T197C-r	CAGGCCCTGATGGCAGACTTCGCAGGC
7-f	LC-Q199C-f	GAAGTCACCCATTGCGGCCTGAGCTCG
7-r	LC-Q199C-r	CGAGCTCAGGCCGCAATGGGTGACTTC
